# LATE-LIFE PLASMA PROTEINS ASSOCIATE WITH PREVALENT AND INCIDENT FRAILTY: A PROTEOMIC ANALYSIS

**DOI:** 10.1093/geroni/igad104.2242

**Published:** 2023-12-21

**Authors:** Fangyu Liu, Thomas Austin, Jennifer Schrack, Jeremy Walston, Rasika Mathias, Josef Coresh, Keenan Walker

**Affiliations:** Johns Hopkins Bloomberg School of Public Health, Baltimore, Maryland, United States; University of Washington, Seattle, Washington, United States; Johns Hopkins University, Baltimore, Maryland, United States; Johns Hopkins University, Baltimore, Maryland, United States; Johns Hopkins University, Baltimore, Maryland, United States; Johns Hopkins University, Baltimore, Maryland, United States; National Institute on Aging, National Institutes of Health, Baltimore, Maryland, United States

## Abstract

Proteomic approaches have unique advantages in the identification of biological pathways that influence physical frailty, a multifactorial geriatric syndrome predictive of adverse health outcomes in older adults. To date, few proteomic studies included prefrailty as a separate state or follow the participants for incident frailty. We evaluated 4955 plasma proteins (log 2-transformed and standardized) measured using SomaScan in the Atherosclerosis Risk in Community (ARIC) study. We used multinomial logistic regression models for the cross-sectional associations of the proteins with prefrailty and frailty as two distinct states (N=3838), and logistic regression models for the longitudinal associations with incident frailty over the 6-year follow-up (N=1708). After adjusting for demographics, health indicators, and comorbidities, 136 and 186 proteins were cross-sectionally associated with prefrailty and frailty, respectively, after Bonferroni correction (p< 1.01x10-5); 24 and 84 of which were replicated in the Cardiovascular Health Study (CHS) using the same models (FDR p< 0.05). Notably, higher odds of prefrailty and frailty were observed with higher concentrations of growth differentiation factor 15 (GDF15; OR[discovery]=1.50 and 2.17, respectively), transgelin (TAGLN; OR[discovery]=1.43 and 2.43, respectively), and insulin-like growth factor-binding protein 2 (IGFBP2; OR[discovery]=1.43 and 2.08, respectively), and lower concentration of growth hormone receptor (GHR, OR[discovery]=0.70 and 0.47, respectively). Longitudinally, triggering receptor expressed on myeloid cells 1 (TREM1) predicted incident frailty (OR[discovery]=1.64). Pathway and upstream regulator analyses revealed that mechanisms related to lipid metabolism, axonal guidance, inflammation and cell senescence became dysregulated in the prefrail stage and persisted into frail stage, offering new insights into frailty etiology and targets for early intervention.

